# FGF1 Suppresses Allosteric Activation of β3 Integrins by FGF2: A Potential Mechanism of Anti-Inflammatory and Anti-Thrombotic Action of FGF1

**DOI:** 10.3390/biom14080888

**Published:** 2024-07-23

**Authors:** Yoko K. Takada, Xuesong Wu, David Wei, Samuel Hwang, Yoshikazu Takada

**Affiliations:** 1 Department of Dermatology, Research III Suite 3300, UC Davis School of Medicine, Sacramento, CA 95817, USA; yoktakada@ucdavis.edu (Y.K.T.); xswu@ucdavis.edu (X.W.); dtwei@ucdavis.edu (D.W.); sthwang@ucdavis.edu (S.H.); 2 Department of Biochemistry and Molecular Medicine, Research III Suite 3300, UC Davis School of Medicine, Sacramento, CA 95817, USA

**Keywords:** integrin, FGF1, FGF2, anti-inflammatory action, anti-thrombotic action

## Abstract

Several inflammatory cytokines bind to the allosteric site (site 2) and allosterically activate integrins. Site 2 is also a binding site for 25-hydroxycholesterol, an inflammatory lipid mediator, and is involved in inflammatory signaling (e.g., TNF and IL-6 secretion) in addition to integrin activation. FGF2 is pro-inflammatory and pro-thrombotic, and FGF1, homologous to FGF2, has anti-inflammatory and anti-thrombotic actions, but the mechanism of these actions is unknown. We hypothesized that FGF2 and FGF1 bind to site 2 of integrins and regulate inflammatory signaling. Here, we describe that FGF2 is bound to site 2 and allosterically activated β3 integrins, suggesting that the pro-inflammatory action of FGF2 is mediated by binding to site 2. In contrast, FGF1 bound to site 2 but did not activate these integrins and instead suppressed integrin activation induced by FGF2, indicating that FGF1 acts as an antagonist of site 2 and that the anti-inflammatory action of FGF1 is mediated by blocking site 2. A non-mitogenic FGF1 mutant (R50E), which is defective in binding to site 1 of αvβ3, suppressed β3 integrin activation by FGF2 as effectively as WT FGF1.

## 1. Introduction

Integrins are a superfamily of cell-surface receptors that bind to extracellular matrix (ECM) (e.g., vitronectin, fibronectin, and collagen), cell surface proteins (e.g., VCAM-1, and ICAM-1), and soluble proteins (e.g., growth factors) [1]. Currently, 18 α and 8 β subunits, which generate 25 heterodimers, are known. Integrins are expressed on a wide range of cell types and play important roles in normal biology (e.g., development, wound healing) and in the pathogenesis of diseases (e.g., thrombosis, cancer metastasis). Integrins induce signals inside the cells (outside-in signals) upon binding to ligands [2].

It has been reported that antagonists to integrin αvβ3 suppressed FGF2-induced angiogenesis and tumor growth, suggesting that integrins are required for FGF signaling (FGF-integrin crosstalk) [3]. Current models of this crosstalk suggest that integrin αvβ3 binds to ECM, and FGFs bind to their cognate FGF receptors [4]. We have shown that FGF1 and FGF2 bind to integrin αvβ3 by virtual screening of a protein data bank (PDB) using docking simulation with the integrin headpiece as a target [5,6]. Docking simulation predicts that FGF1 and FGF2 bind to the classical ligand (RGD)-binding site of αvβ3 (site 1) [5,6]. FGF1 binding to integrin αvβ3 induced integrin αvβ3-FGF1-FGFR1 ternary complex formation [7]. The FGF1 mutant defective in integrin binding due to a point mutation in the predicted integrin-binding site (Arg50 to Glu, R50E) did not bind to integrin αvβ3 but still bound to FGFR and heparin and was defective in inducing sustained ERK1/2 activation, ternary complex formation, and inducing mitogenesis [5]. The R50E FGF1 mutant was defective in inducing angiogenesis and suppressed angiogenesis induced by WT FGF1 (dominant-negative effect) [8]. We obtained similar results in FGF2 [6]. The FGF2 mutants defective in integrin binding were defective in signaling and ternary complex formation and acted as dominant-negative antagonists [6]. It has been reported that FGF2 and integrin α6β1 are important for maintaining the pluripotency of human pluripotent stem cells (hPSCs). It has recently been reported that integrin α6β1-FGF2-FGFR ternary complex formation is critical for maintaining the pluripotency of hPSCs [9]. 

**Figure 1:**
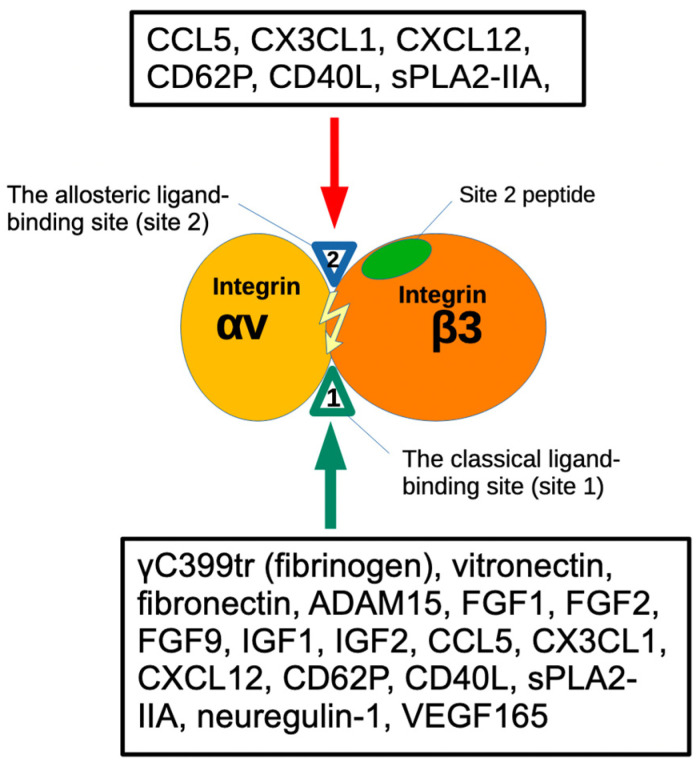
Binding of ligands to the classical-ligand binding site (site 1) and the allosteric binding site (site 2) of αvβ3. Fibrinogen γ-chain C-terminal peptide (γC399tr) and ADAM15 [20] specifically bind to the classical ligand binding site (site1) of αvβ3. FGF9 [21], the heparin-binding site of VEGF165 [22]. FGF1 [5], FGF2 [6], IGF1 [23], IGF2 [24], neuregulin [25], pro-inflammatory CX3CL1 [18], CCL5 [14], CD40L [25,26], CD62P binds to site 2 and activate αvβ3 [17]. Site 2-derived peptides bind to these ligands and suppress integrin activation [15].

**Figure 2:**
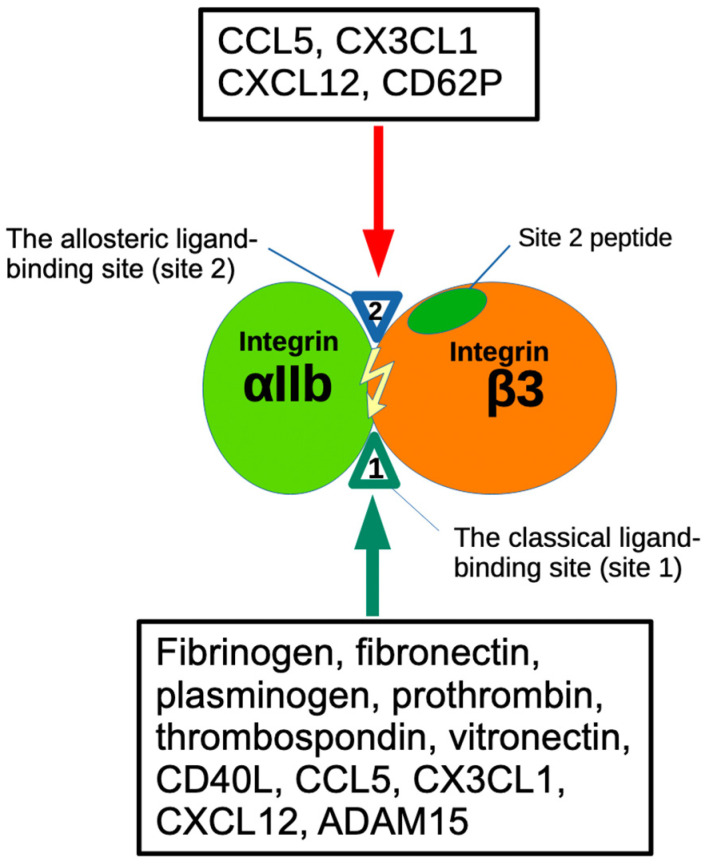
The binding of ligands to the Fibrinogen γ-chain C-terminal peptide (γC390-411) and ADAM15 [27] specifically occurs at the classical ligand binding site (site1) of αIIbβ3. Several inflammatory chemokines (CCL5, CX3CL1, CXCL12) [14] and pro-inflammatory CD62P bind to site 2 and activate αIIbβ3 [17]. Site 2-derived peptides bind to these ligands and suppress integrin activation [15].

It has been well established that integrin activation can be mediated by signals from inside the cells (inside-out signaling) [10,11]. We discovered, however, that several pro-inflammatory proteins such as CX3CL1 (fractalkine) [12]; CXCL12 (SDF-1) [13,14]; Rantes (CCL5) [14]; and secreted phospholipase A2 type IIA (sPLA2-IIA) [15], CD40L [16], and P-selectin [17] activated integrins including αvβ3 independent of inside-out signaling. We found that this activation is induced by ligand binding to the allosteric ligand-binding site (site 2) of integrins. Site 2 is distinct from site 1 and is on the opposite side of site 1 in the integrin headpiece [13,14,18] (Figure 1 and Figure 2). We showed that cyclic peptides from site 2 bound to these allosteric activators and suppressed integrin activation, indicating that allosteric activators are required to bind to site 2 for integrin activation. Since allosteric activation is induced by inflammatory cytokines, there should be a link between allosteric integrin activation and inflammatory signaling. It has been reported that the pro-inflammatory lipid mediator, 25-hydroxycholesterol, binds to site 2 of integrins and activates integrins and induces inflammatory signals (e.g., secretion of IL-6 and TNF) [19], which verifies the role of site 2 in allosteric integrin activation and inflammatory signals in inflammation. 

Activation of αIIbβ3 is a key event that triggers platelet aggregation upon platelet activation by inducing αIIbβ3 binding to fibrinogen, leading to bridge formation between platelets [10,11]. Activation of αIIbβ3 is mediated exclusively by inside-out signaling induced by platelet agonists (e.g., thrombin, ADP, and collagen) [28]. We have discovered that several cytokines, including those stored in platelet granules (CCL5, CXCL12, CD40L, and P-selectin) activate αIIbβ3 by binding to site 2 in an allosteric manner [14,17,25]. We hypothesize that inflammatory cytokines stored in platelet granules play an important role in activating αIIbβ3. 

FGF2 induces the expression of a wide repertoire of inflammation-related genes in endothelial cells, including pro-inflammatory cytokines/chemokines and their receptors, endothelial cell adhesion molecules, and components of the prostaglandin pathway [29]. FGF2 expression is enhanced in endothelial precursor cells in deep vein thrombosis [30], suggesting that FGF2 is pro-thrombotic. FGF2 is stored in platelet granules and rapidly transported to the surface upon platelet activation. The mechanism of pro-inflammatory or pro-thrombotic action of FGF2 is unknown. 

FGF1 belongs to the same subfamily as FGF2 (FGF1 subfamily) and is not stored in platelet granules. Previous studies showed that FGF1 prevented the development of several inflammatory diseases [31]. Also, FGF1 has been shown to lower blood glucose levels in diabetic mice, but the mechanism of this action is unknown [32]. FGF1 is shown to be cardioprotective (anti-thrombotic) and blocking FGF1 synthesis by activating FGF1 promoter methylation exacerbated deep vein thrombosis [33]. However, the mechanism of the anti-inflammatory and cardioprotective action of FGF1 is unknown. We studied how FGF1 and FGF2 regulate β3 integrins. We describe here that FGF2 activated β3 integrins by binding to site 2. FGF1 also bound to site 2 but did not activate β3 integrins and instead suppressed integrin activation induced by FGF2, indicating that FGF1 acts as an antagonist of site 2. We propose a model in which pro-inflammatory and pro-thrombotic actions of FGF2 are mediated by binding to site 2, and anti-inflammatory and anti-thrombotic action of FGF1 is mediated by inhibiting site 2-mediated β3 integrin activation and inflammatory signaling by FGF2. Non-mitogenic FGF1 R50E also suppressed the activation of β3 integrins by FGF2 in cell-free conditions, suggesting that FGF1 R50E has therapeutic potential.

## 2. Materials and Methods

### 2.1. Materials

The truncated fibrinogen γ-chain C-terminal domain (γC399tr) was generated as previously described [34]. Fibrinogen γ-chain C-terminal residues 390-411 cDNA encoding (6 His tagged) [HHHHHH]NRLTIGEGQQHHLGGAKQAGDV] was conjugated with the C-terminus of GST (designated γC390-411) in pGEXT2 vector (BamHI/EcoRI site). The protein was synthesized in E. coli BL21 and purified using glutathione affinity chromatography. The protein was synthesized in E. coli BL21 and purified using glutathione affinity chromatography. FGF1 [5] and FGF2 [6] were synthesized as previously described. 

Cyclic β3 site 2 peptide fused to GST-The 29-mer cyclic β3 site 2 peptide C260-RLAGIV[QPNDGSHVGSDNHYSASTTM]C288 (C273 is changed to S) was synthesized by inserting oligonucleotides encoding this sequence into the BamHI/EcoRI site of pGEX-2T vector. The positions of Cys residues for disulfide linkage were selected by using Disulfide by Design-2 (DbD2) software v2.12 (http://cptweb.cpt.wayne.edu/DbD2/) [35]. It predicted that mutating Gly260 and Asp288 to Cys disulfide-linked cyclic site 2 peptide of β3 does not affect the conformation of the original site 2 peptide sequence QPNDGSHVGSDNHYSASTTM in the 3D structure. We found that the cyclic site 2 peptide bound to CX3CL1 and sPLA2-IIA to a similar extent to non-cyclized β3 site 2 peptides in ELISA-type assays. We designed the corresponding cyclic β1 peptide (C268-KLGGIVLPNDGQSHLENNMYTMSHYYC295, 28- mer cyclic β1 peptide) in which C281 is converted to S. We synthesized the proteins in BL21 cells and purified using glutathione-Sepharose affinity chromatography. 

Site-directed mutagenesis was performed using the QuikChange method [36]. The presence of the mutations was verified by DNA sequencing.

### 2.2. Activation of Soluble αIIbβ3 and αvβ3 by FGF2

ELISA-type binding assays were performed as described previously [13]. Briefly, wells of 96-well Immulon 2 microtiter plates (Dynatech Laboratories, Chantilly, VA, USA) were coated with 100 μL 0.1 M PBS containing γC390-411 for αIIbβ3 and γC399tr for αvβ3 for 2 h at 37 °C. The remaining protein-binding sites were blocked by incubating with PBS/0.1% BSA for 30 min at room temperature. After washing with PBS, soluble recombinant αIIbβ3 or αvβ3 (1 μg/mL) in the presence or absence of FGF1 and/or FGF2 was added to the wells and incubated in Hepes–Tyrodes buffer (10 mM HEPES, 150 mM NaCl, 12 mM NaHCO_3_, 0.4 mM NaH_2_PO_4_, 2.5 mM KCl, 0.1% glucose, 0.1% BSA) with 1 mM CaCl_2_ for 1 h at room temperature. After unbound αIIbβ3 or αvβ3 was removed by rinsing the wells with binding buffer, bound αIIbβ3 or αvβ3 was measured using anti-integrin β3 mAb (AV-10) followed by HRP-conjugated goat anti-mouse IgG and peroxidase substrates. 

### 2.3. Activation of Integrin αIIbβ3 on the Cell Surface by FGF2

CHO cells that express recombinant αIIbβ3 (αIIbβ3-CHO) were cultured to nearly confluent in DMEM/10% FCS. Cells were resuspended with DMEM/0.02% BSA and incubated for 30 min at room temperature to block protein-binding sites. Cells were then incubated with WT FGF2 or mutants for 5 min at room temperature and then incubated with FITC-labeled γC390-411 for 15 min at room temperature. Cells were washed with PBS/0.02% BSA and analyzed by FACSCalibur (Becton Dickinson, Mountain View, CA, USA). For blocking experiments, FGF2 was preincubated with Fc-β3 peptide for 30 min at room temperature.

### 2.4. Binding of Site 2 Peptide to FGF1 and FGF2

ELISA-type binding assays were performed as described previously [12]. Briefly, wells of 96-well Immulon 2 microtiter plates (Dynatech Laboratories, Chantilly, VA, USA) were coated with FGF1 or FGF2 (10 μg/mL) in 100 μL 0.1 M PBS for 2 h at 37 °C. The remaining protein-binding sites were blocked by incubating with PBS/0.1% BSA for 30 min at room temperature. After washing with PBS, GST-β3 peptide (100 μg/mL) was added to the wells and incubated in PBS for 1 h at room temperature. After the unbound site 2 peptide was removed by rinsing the wells with PBS, the bound site 2 peptide was measured using an anti-GST antibody and HRP-conjugated anti-mouse IgG.

### 2.5. Docking Simulation

Docking simulation of the interaction between FGF2 and integrin αvβ3 (closed headpiece form, PDB code 1JV2) was performed using AutoDock3, as described previously [37]. We used the headpiece (residues 1–438 of αv and residues 55–432 of β3) of αvβ3 (closed form, 1JV2.pdb). Cations were not present in integrins during docking simulation, as in the previous studies using αvβ3 (open headpiece form, 1L5G.pdb) [5,38]. The ligand is presently compiled to a maximum size of 1024 atoms. Atomic solvation parameters and fractional volumes were assigned to the protein atoms by using the AddSol utility, and grid maps were calculated by using the Auto Grid utility in AutoDock 3.05. A grid map with 127 × 127 × 127 points and a grid point spacing of 0.603 Å included the headpiece of αvβ3. Kollman ‘united-atom’ charges were used. AutoDock 3.05 uses a Lamarckian genetic algorithm (LGA) that couples a typical Darwinian genetic algorithm for global searching with the Solis and Wets algorithm for local searching. The LGA parameters were defined as follows: the initial population of random individuals had a size of 50 individuals; each docking was terminated with a maximum number of 1 × 10^6^ energy evaluations or a maximum number of 27,000 generations, whichever came first; mutation and cross-over rates were set at 0.02 and 0.80, respectively. An elitism value of 1 was applied, which ensured that the top-ranked individual in the population always survived into the next generation. A maximum of 300 iterations per local search were used. The probability of performing a local search on an individual was 0.06, whereas the maximum number of consecutive successes or failures before doubling or halving the search step size was 4.

### 2.6. Statistical Analysis

Treatment differences were tested using ANOVA and Tukey multiple comparison tests to control the global type I error using Prism 10 (Graphpad Software, Boston, MA, USA).

## 3. Results

### 3.1. FGF1 and FGF2 Bind to the Classical Binding Site (Site 1) of αIIbβ3 

**Figure 3:**
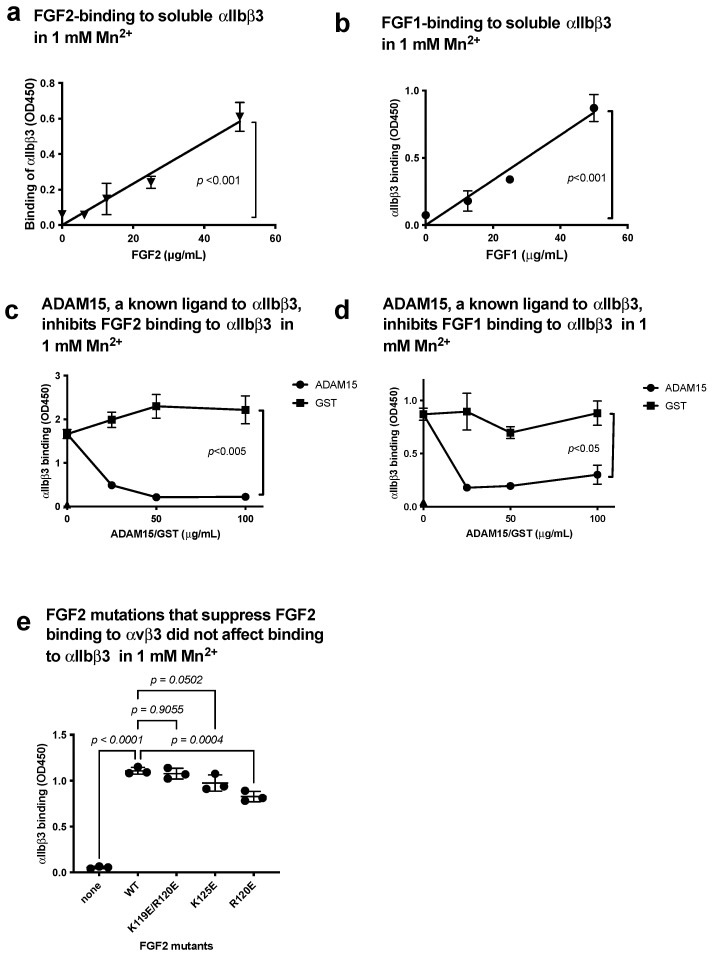
FGF2 and FGF1 bind to site 1 of αIIbβ3. (**a**) FGF2 binds to soluble αIIbβ3. Soluble αIIbβ3 was incubated with immobilized FGF2 in 1 mM Mn^2+^. Bound αIIbβ3 was quantified using anti-β3 and HRP-conjugated anti-mouse IgG. (**b**) FGF1 binds to soluble αIIbβ3. The binding of soluble αIIbβ3 to immobilized FGF1 was measured as in (**a**), except FGF1 was used instead of FGF2. (**c**,**d**) Inhibition of FGF1/FGF2 binding to soluble αIIbβ3 by ADAM15, another ligand to αIIbβ3. Wells of 96-well microtiter plate were coated with FGF2 (**c**) or FGF1 (**d**) and incubated with soluble αIIbβ3 in the presence of ADAM15 disintegrin domain fused to GST or control GST in 1 mM Mn^2+^. (**e**) Effect of FGF2 mutation that blocked binding to αvβ3 (site 1) on binding to αIIbβ3. FGF2 mutants defective in binding to αvβ3 (site 1) were tested for their ability to bind to αIIbβ3 in 1 mM Mn^2+^. The data are shown as means +/− SD in triplicate experiments.

FGF1 and FGF2 are known to bind to site 1 of αvβ3 [5,6], but it is unclear if FGF1 and FGF2 bind to αIIbβ3. We studied if soluble αIIbβ3 bound to FGF2 and FGF1 in ELISA-type binding assays. We detected the binding of soluble αIIbβ3 to FGF1 and FGF2 in a dose-dependent manner in 1 mM Mn^2+^ (Figure 3a,b). To show the specificity of FGF2 and FGF1 binding to αIIbβ3, we tested if FGF2 and FGF1 compete with known ligands for αIIbβ3 for binding to αIIbβ3. The disintegrin domain of ADAM15, a specific ligand for αIIbβ3 [27], potently suppressed FGF2 and FGF1 binding to αIIbβ3 (Figure 3c,d), indicating that the binding of FGF2 and FGF1 to αIIbβ3 is specific. We previously reported that FGF2 mutants were defective in binding to site 1 of αvβ3 (the K119E/R120E and K125E mutants) [6]. The FGF2 mutants were defective in binding to αvβ3 bound to αIIbβ3 in 1 mM Mn^2+^ (Figure 3e), indicating that FGF2 binds to site 1 of αIIbβ3 and αvβ3 in a different manner. 

### 3.2. FGF2 Activates Soluble Integrin αIIbβ3 by Binding to the Allosteric Site (Site 2)

**Figure 4:**
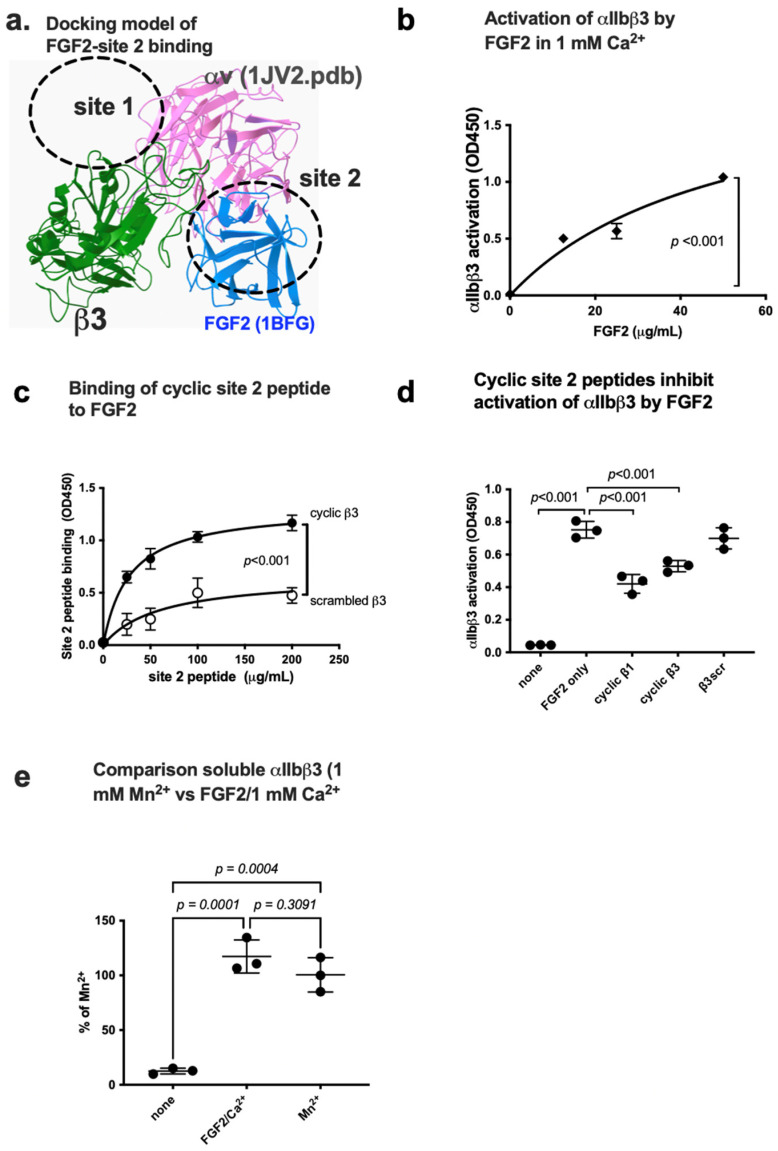
FGF2 binds to site 2 of integrin αIIbβ3 and activates αIIbβ3. (**a**) Docking simulation of the interaction between FGF2 (1BFG.pdb) and αvβ3 (closed headpiece form, 1JV2.pdb). (**b**) FGF2 activates αIIbβ3. The fibrinogen fragment (γC390-411) fused to GST, a specific ligand to αIIbβ3, was immobilized to wells of a 96-well microtiter plate and incubated with soluble αIIbβ3 (1 μg/mL) and bound αIIbβ3 was measured in 1 mM Ca^2+^ (to keep integrins inactive). (**c**) Binding of FGF2 to site 2 peptide of β3. FGF2 was immobilized to wells of a 96-well microtiter plate and incubated with cyclic site 2 peptide fused to GST or control scrambled β3 peptide, and the bound peptide was measured using anti-GST. (**d**) Cyclic site 2 peptides inhibit activation of αIIbβ3 by FGF2. Activation of αIIbβ3 was measured as described in (**b**). The concentrations used were 20 μg/mL (FGF2) and 100 μg/mL (site 2 peptides). Bound integrin was measured using anti-β3 mAb. (**e**) FGF2 activates soluble αIIbβ3 to an extent similar to that of 1 mM Mn^2+^. Activation of αIIbβ3 was measured as described in (**b**) using 1 mM Mn^2+^ or FGF2 (50 μg/mL). The data are normalized with 1 mM Mn^2+^ as 100%. The data are shown as means +/− SD in triplicate experiments. ANOVA using Prism 10 was used for statistical analysis (*n* = 3).

We previously showed that several inflammatory cytokines stored in platelet granules activate αIIbβ3 upon platelet activation by binding to site 2. It is, however, unknown if FGF2 binds to site 2 and activates αIIbβ3. We performed a docking simulation of the interaction between closed headpiece integrin αvβ3 (1JV2.pdb) and FGF2 using Autodock3. The 3D structure of closed headpiece αvβ3 structure (1JV2.pdb) was used instead of αIIbβ3 since closed headpiece conformation is well defined in αvβ3, but not in αIIbβ3. The simulation predicts that FGF2 binds to site 2 of αvβ3 (docking energy −20.5 kcal/mol) (Figure 4a). 

We found that FGF2 activated integrin αIIbβ3 in 1 mM Ca^2+^ in cell-free conditions in ELISA-type integrin activation assays. Wells of 96-well microtiter plate was coated with a fibrinogen fragment (γC390-411), a specific ligand for αIIbβ3, and incubated with soluble integrin αIIbβ3 in 1 mM Ca^2+^ in the presence of FGF2. FGF2 enhanced the binding capacity of soluble αIIbβ3 to γC390-411 in a dose-dependent manner (Figure 4b). 

Several amino acid residues involved in site 2 of β3 are predicted to be involved in this interaction (Table 1). Consistently, we found that cyclic peptides derived from site 2 of β3 bound to FGF2 at higher levels than control scrambled β3 peptide, indicating that FGF2 binds to site 2 to activate αIIbβ3 (Figure 4c). We found that cyclic site 2 peptides from integrin β1 or β3 suppressed αIIbβ3 activation by FGF2 (Figure 4d), indicating that activation of αIIbβ3 by FGF2 requires FGF2 binding to site 2. It has been assumed that 1 mM Mn^2+^ fully activates integrins [39,40,41,42]. We compared the levels of activation of soluble αIIbβ3 by FGF2 with that of 1 mM Mn^2+^ as a standard integrin activator. The level of activation of αIIbβ3 by FGF2 was comparable to that of 1 mM Mn^2+^ (Figure 4e).

We needed high concentrations of FGF2 for activation of soluble integrins in cell-free conditions. In our preliminary studies, we detected activation of cell surface αIIbβ3 at 1–10 ng/mL FGF2 (Supplemental Figure S1). Since FGF2 can be highly concentrated on the cell surface by binding to cell surface proteoglycans, allosteric activation of integrins by FGF2 is biologically relevant.

### 3.3. Point Mutations of the Predicted Site 2-Binding Interface in FGF2 Block FGF2-Mediated Activation of αIIbβ3

**Figure 5:**
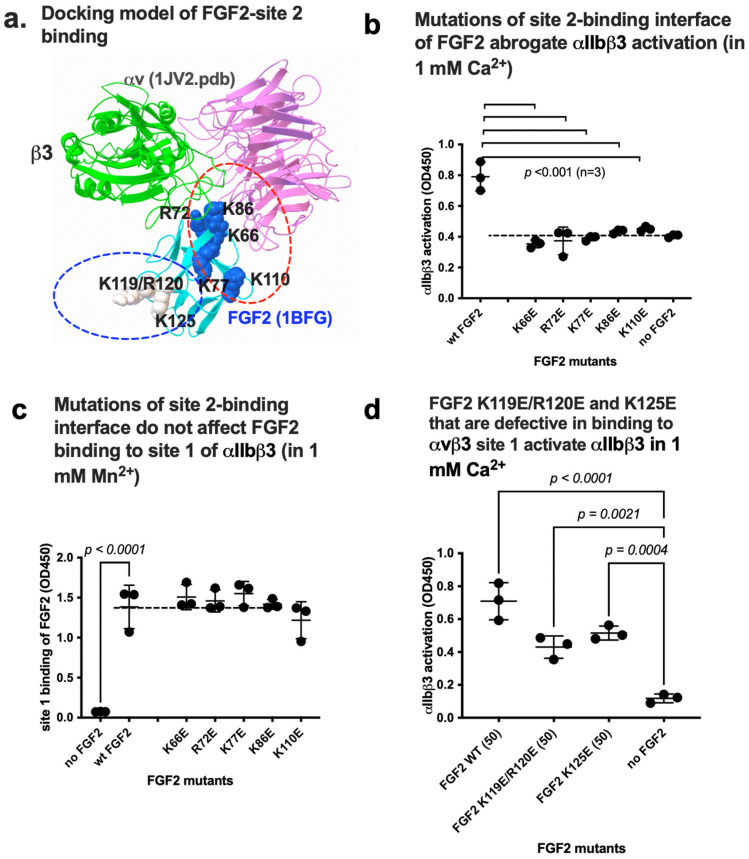
Point mutations in site 2-binding interface of FGF2 effectively reduce activation of integrin αIIbβ3 by FGF2. (**a**) Positions of amino acid residues involved in site 2 binding predicted by docking simulation. K119E/R120E and K125E mutations suppressed FGF2 binding to integrin site 1 of αvβ3 and thereby suppressed FGF2 mitogenicity [6]. Arg72, Lys77, Lys86, and Lys110 are in the predicted site 2-binding interface of FGF2. (**b**) Mutations in the site 2 binding interface of FGF2 blocked activation of αIIbβ3 in 1 mM Ca^2+^. (**c**) The point mutations in the predicted site 2-binding site of FGF2 did not affect FGF2 binding to site 1 in 1 mM Mn^2+^. The results indicate that site 1 and site 2-binding sites in FGF2 are distinct. (**d**) FGF2 mutants of K119E/R120E and K125E still activate αIIbβ3. The data are shown as means +/− SD in triplicate experiments.

To further show how FGF2 binds to site 2 of αIIbβ3, we generated FGF2 mutants defective in site 2 binding. The docking simulation of interaction between FGF2 (1BFG.pdb) and αvβ3 (1JV2.pdb) predicts that amino acid residues Lys66, Arg72, Lys77, Lys86, and Lys110 of β3 interact with αvβ3 (docking energy −20.5 Kcal/mol) (Figure 5a) (Table 1). We found that mutation of these amino acids to Glu (K66E, K72E, K77E, K86E, and K110E mutations) suppressed integrin activation by FGF2 (Figure 5b), indicating that the docking model is correct and that FGF2 binding to site 2 is required for activation of αIIbβ3. We found that K66E, K72E, K77E, K86E, and K110E mutations did not block FGF2 binding to site 1 of αIIbβ3 in 1 mM Mn^2+^ (Figure 5c), indicating that the effect of the mutations in the site 2-binding site is specific and does not affect site 1 binding. 

### 3.4. FGF2 Activates Soluble Integrin αvβ3 by Binding to the Allosteric Site (Site 2)

**Figure 6:**
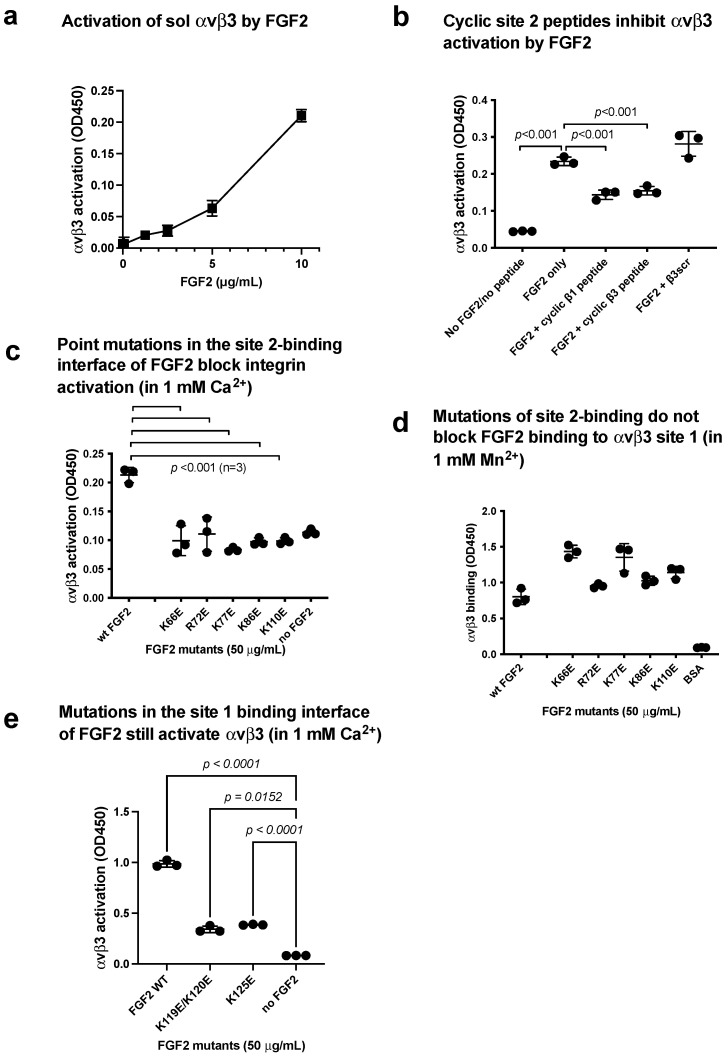
FGF2 binds to site 2 of integrin and αvβ3 and activates integrins (act as an agonist). (**a**) FGF2 activated αvβ3. The fibrinogen fragment (γC399tr), a specific ligand to αvβ3, was immobilized to wells of a 96-well microtiter plate and incubated with soluble αvβ3 (1 μg/mL) in the presence of FGF2, and bound αvβ3 was measured using anti-β3 mAb. The data show that FGF2 activated αvβ3. (**b**) Cyclic site 2 peptides inhibit activation of αvβ3 by FGF2. Activation of αvβ3 was measured as described in (**a**). The concentrations used were 20 μg/mL (FGF2) and 100 μg/mL (site 2 peptides). Bound integrin was measured using anti-β3 mAb. Cyclic site 2 peptides from β1 or β3 suppressed αvβ3 activation by FGF2, but control β3 scrambled peptide did not. (**c**) FGF2 with point mutations in the predicted site 2-binding interface of FGF2 did not activate integrin αvβ3. (**d**) Point mutations in the predicted site 2-binding interface did not affect FGF2 binding to site 1 in 1 mM Mn^2+^. Wells of 96-well microtiter plate were coated with FGF2 WT and mutants and incubated with soluble αvβ3 in 1 mM Mn^2+^. Bound αvβ3 was quantified using anti-β3 and anti-mouse IgG conjugated with HRP. (**e**) Mutations in the site 1 binding interface of FGF2 still activate αvβ3 (in 1 mM Ca^2+^). Activation assays were performed as described in (**a**). The data are shown as means +/− SD in triplicate experiments.

Integrins αIIbβ3 and αvβ3 have a common β3 subunit, but their cellular distribution and biological functions are distinct. The docking simulation of interaction between FGF2 and αvβ3 predicts that FGF2 binds to site 2 (Figure 4a). Cyclic site 2-derived peptide bound to FGF2 to a greater extent than the control scrambled peptide (Figure 4c). We found that FGF2-activated integrin αvβ3 in 1 mM Ca^2+^ in cell-free conditions in ELISA-type integrin activation assays using a fibrinogen fragment (γC399tr), a specific ligand for αvβ3, and soluble integrin αvβ3 in a dose-dependent manner (Figure 6a). We found that cyclic site 2 peptides from integrin β1 or β3 suppressed αvβ3 activation by FGF2 (Figure 6b), indicating that activation of αvβ3 by FGF2 requires FGF2 binding to site 2. 

We found that FGF2 mutants (K66E, K72E, K77E, K86E, and K110E) effectively suppressed integrin αvβ3 activation by FGF2 (Figure 6c), indicating that the binding interface of FGF2 to αvβ3 and αIIbβ3 overlap, and FGF2 binding to site 2 is required for allosteric activation of αvβ3. However, these mutants are still bound to αvβ3 site 1 (Figure 6d). 

The FGF2 mutants (K119E/R120E and K125E) in the integrin-binding interface of FGF2 were defective in signaling and ternary complex formation and acted as dominant-negative antagonists for FGF2 signaling through site 1 of αvβ3 [6]. The FGF2 mutants (K119E/R120E and K125E) seem to bind to αvβ3 site 2 (Figure 6e), αIIbβ3 site 1 (Figure 3e), and αIIbβ3 site 2 (Figure 5d).

### 3.5. FGF1 Binds to Site 2 but Does Not Activate β3 Integrins and Suppresses β3 Integrin Activation Induced by FGF2

**Figure 7:**
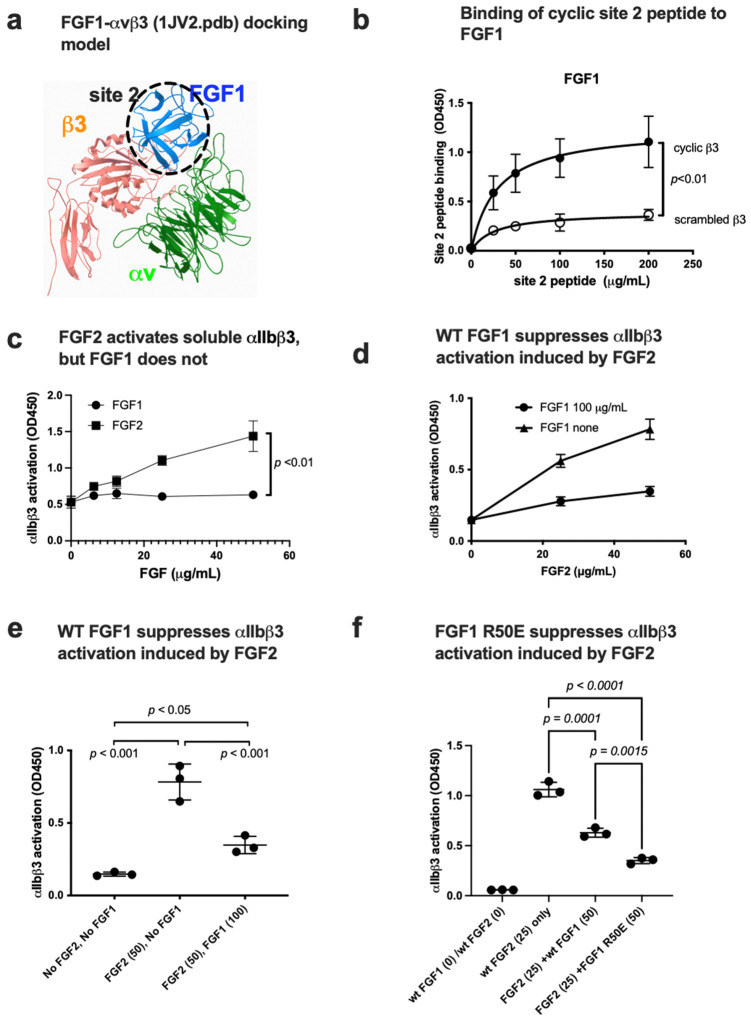
FGF1 binds to site 2 but suppresses FGF2-induced activation of αIIbβ3. (**a**) FGF1 is predicted to bind to site 2. Docking simulation of the interaction between FGF1 and site 2 of close headpiece form of αvβ3 (1JV2.pdb). (**b**) Binding of cyclic site 2 peptide to FGF1. (**c**) FGF1 does not activate soluble αIIbβ3. Wells of 96-well microtiter plate were coated with γC390-411, a specific ligand to αIIbβ3, and incubated with soluble αIIbβ3 in the presence of WT FGF2 or FGF1 in 1 mM Ca^2^. (**d**,**e**). FGF1 suppresses FGF2-induced activation of soluble αIIbβ3. Activation of soluble αIIbβ3 was assayed as described in (**b**). (**f**) Non-mitogenic FGF1 mutant (R50E) suppressed integrin activation by FGF2 at a level comparable to that of WT FGF1 in Ca^2+^.

**Figure 8:**
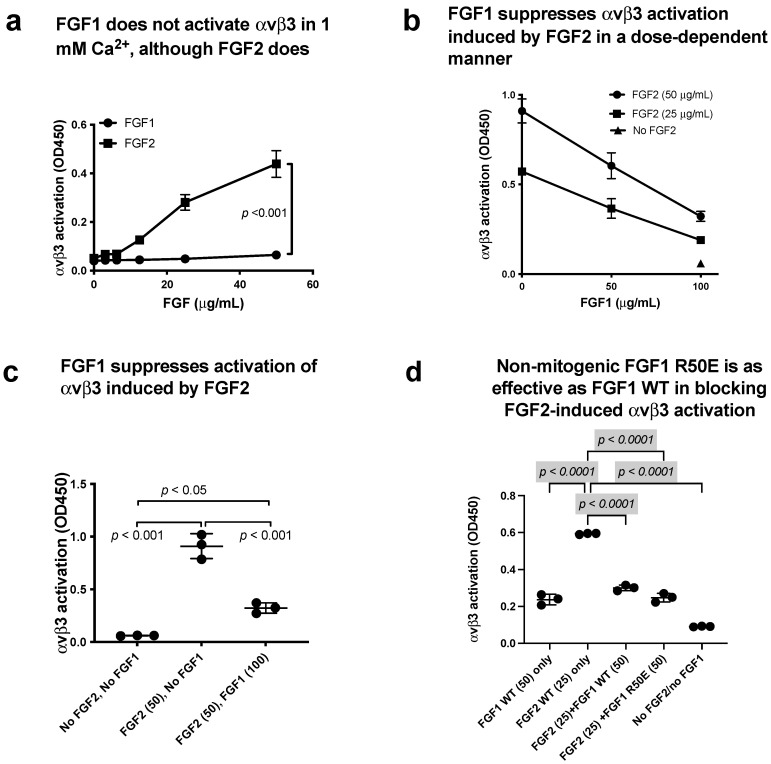
FGF1 binds to site 2 but does not activate αvβ3. FGF1 suppressed integrin activation by FGF2. (**a**) FGF2 allosterically activated soluble αvβ3 in a dose-dependent manner, but FGF1 did not. Wells of 96-well microtiter plate were coated with γC399, a specific ligand for αvβ3, and incubated with soluble αvβ3 in 1 mM Ca^2+^. Bound αvβ3 was quantified using anti-β3. (**b**,**c**) FGF1 inhibits integrin activation by FGF2. Soluble αvβ3 (1 μg/mL) was incubated with immobilized ligand (γC399tr specific to αvβ3) in the presence of FGF2 and/or FGF1 in 1 mM Ca^2+^. (**d**) Non-mitogenic FGF1 mutant (R50E) suppressed integrin activation by FGF2 at a level comparable to that of WT FGF1 in Ca^2+^.

Docking simulation of the interaction between FGF1 and closed headpiece αvβ3 (1JV2.pdb) predicts that FGF1 binds to site 2 of αvβ3 (docking energy −20.1 kcal/mol) (Figure 7a). Several amino acid residues involved in site 2 of β3 are predicted to be involved in this interaction (Table 2). Consistently, we found that cyclic peptides derived from site 2 of β3 bound to FGF1 at higher levels than control scrambled β3 peptide, indicating that FGF1 binds to site 2 of β3 integrins (Figure 7b). Thus, we expected that FGF1 also activates β3 integrins. Unexpectedly, FGF1 did not activate αIIbβ3 (Figure 7c) or αvβ3 (Figure 8a) at all under the conditions in which FGF2 activated these integrins. Notably, FGF1 suppressed αIIbβ3 activation by FGF2 (Figure 7d,e) or αvβ3 activation by FGF2 (Figure 8b,c) in a dose-dependent manner, suggesting that FGF1 binds to site 2 and acts as a competitive inhibitor of allosteric activation by FGF2 (site 2 antagonist). 

### 3.6. Non-Mitogenic FGF1 R50E Mutant Suppressed FGF2-Induced Activation of β3 Integrins

We found that non-mitogenic FGF1 R50E suppressed FGF2-induced αIIbβ3 activation (Figure 7f) and activation of αvβ3 by FGF2 (Figure 8d), as effectively as WT FGF1. These findings suggest that Arg-50 is not involved in site 2 binding (amino acid residues involved in site 1 and site 2 binding are not identical).

## 4. Discussion

The present study establishes that FGF1 and FGF2 bind to site 1 of αIIbβ3, indicating that αIIbβ3 is a new receptor for FGF1 and FGF2. αIIbβ3 is known as a receptor for ECM proteins (e.g., fibronectin, fibrinogen, plasminogen, prothrombin, thrombospondin and vitronectin) in addition to CD40L [43,44]. We recently showed that αIIbβ3 binds to several pro-inflammatory proteins (e.g., CX3CL1, CXCL12, CCL5 [14], CD40L [25], and CD62P [17]). These findings suggest that αIIbβ3 may be as promiscuous as integrin αvβ3. 

Activation of αIIbβ3 is a key event in platelet aggregation and subsequent thrombus formation. We propose that several inflammatory proteins (FGF2, CX3CL1, CXCL12, CCL5, CD40L, and CD62P) in platelet granules play a critical role in allosteric αIIbβ3 activation and subsequently induce platelet aggregation and thrombus formation. In the present study, we found that FGF2 bound to site 2 and allosterically activated αIIbβ3, suggesting that FGF2 is involved in platelet aggregation by triggering αIIbβ3 activation in an allosteric manner. These findings are consistent with the recent report that FGF2 expression is enhanced in endothelial precursor cells in deep vein thrombosis [30]. We propose that inflammatory cytokines stored in platelet granules, including FGF2, may play a critical role in triggering platelet aggregation by activating αIIbβ3 in an allosteric manner.

**Figure 9:**
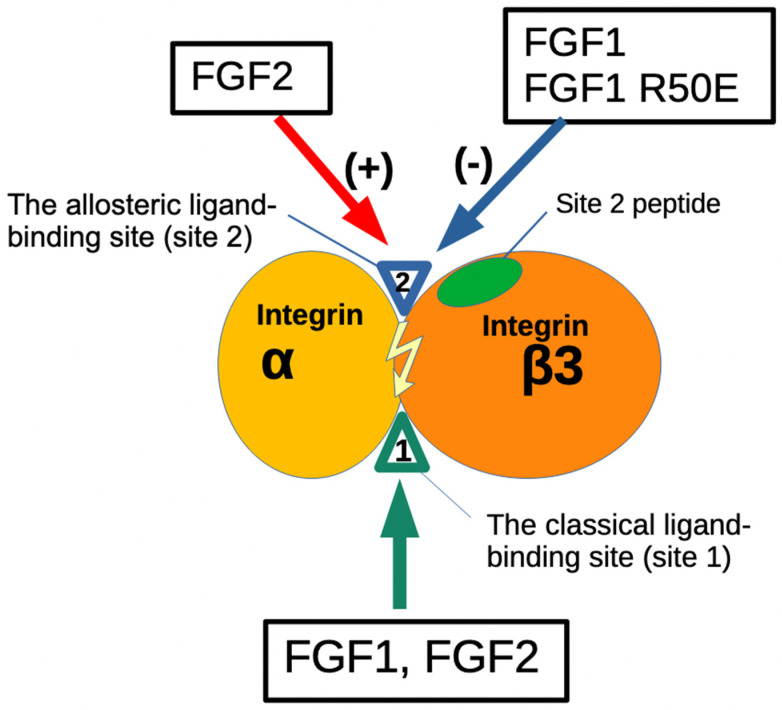
Agonistic action of FGF2 and antagonistic action of FGF1 to the allosteric site (site 2) of β3 integrins. The present study showed that FGF1 and FGF2 bind to the site 1 of β3 integrins. FGF2 (stored in platelet granules) binds to site 2 as well and induces allosteric activation of β3 integrins, leading to platelet aggregation and pro-inflammatory signals. In contrast, FGF1 binds to site 2 but suppresses β3 integrin activation induced by FGF2. This is a potential mechanism of anti-thrombotic action or anti-inflammatory action of FGF1. Non-mitogenic FGF1 R50E mutant is comparable to WT FGF1 in inhibiting FGF2-induced activation of β3 integrins. FGF1 R50E has potential as an anti-thrombotic and anti-inflammatory agent.

The present study found that FGF1 bound to site 2 of αIIbβ3 but did not activate αIIbβ3. Instead, FGF1 suppressed activation of αIIbβ3 induced by FGF2, indicating that FGF1 acts as an antagonist of site 2 (Figure 9). FGF1 is shown to be cardioprotective (anti-thrombotic) and blocking FGF1 synthesis by activating FGF1 promoter methylation exacerbated deep vein thrombosis [33]. However, the mechanism of the anti-thrombotic action of FGF1 is unclear. We propose that FGF1’s anti-thrombotic action may be mediated by blocking αIIbβ3 activation by FGF2 from platelet granules. Therefore, we will need to study inflammatory signaling through site 2 in future studies. 

There is growing recognition of the critical role of platelets in inflammation and immune responses [45,46]. Recent studies have indicated that anti-platelet medications may reduce mortality from infections and sepsis, which suggests the possible clinical relevance of modifying platelet responses to inflammation. Platelets release numerous inflammatory mediators that have no known role in hemostasis [47]. Many of these mediators modify leukocyte and endothelial responses to a range of different inflammatory stimuli. Additionally, platelets form aggregates with leukocytes and form bridges between leukocytes and endothelium [46], largely mediated by platelet CD62P. Thus, the present finding that pro-inflammatory FGF2 allosterically activates αIIbβ3 suggests that FGF2 stored in platelet granules also plays a role in the inflammatory actions of platelets. FGF2 may be highly concentrated on the platelet surface upon platelet activation. Also, FGF1 may exert anti-inflammatory action by suppressing αIIbβ3 activation in platelets and subsequently blocking platelet activation and thrombosis. 

The present study showed that FGF2 also bound to site 2 of αvβ3 and activated αvβ3, indicating that FGF2 acts as an agonist of site 2 of αvβ3 (Figure 9). In contrast, FGF1 bound to site 2 of αvβ3 but did not activate αvβ3. Instead, FGF1 suppressed activation of αvβ3 induced by FGF2, indicating that FGF1 acts as an antagonist of site 2 of αvβ3, as in the case of αIIbβ3. Previous studies showed that FGF1 remarkably lowered levels of several serum inflammatory cytokines and impeded the inflammatory response [32,48]. FGF1 significantly prevented the development of nonalcoholic fatty liver disease (NAFLD) and diabetic nephropathy (DN) [48,49]. These findings suggest that FGF1 is anti-inflammatory [31]. Also, FGF1 has been shown to lower blood glucose levels in diabetic mice, but the mechanism of this action is unknown [32]. FGF1 may act through the central nervous system [50] or through FGFR in the adipose tissue [51]. We propose that FGF1’s anti-inflammatory actions may be mediated by blocking inflammatory signals through site 2 of αvβ3 or other integrins. 

Since WT FGF1 is a potent mitogen and cannot be used for a long time as a therapeutic, non-mitogenic FGF1 has been sought. Deletion of N-terminal residues of FGF1 (amino acid residues 21-27), which lacks nuclear translocation signal, has been shown to be non-mitogenic and still has anti-inflammatory activity [52]. This N-terminal truncated FGF1-induced angiogenesis [53]. It is unknown how the deletion of N-terminal residues of FGF1 makes FGF1 non-mitogenic. FGF1 R50E has been well characterized as non-mitogenic FGF1 [5]. We showed that FGF1 R50E suppressed tumorigenesis and angiogenesis in vivo [5], although it has been reported that R50E showed short-term mitogenicity [54]. It is thus unlikely that the anti-inflammatory and glucose-lowering action of FGF1 R50E requires FGFR. We propose that FGF1 R50E is a candidate non-mitogenic antagonist of site 2. 

It has been well documented that antagonists to αIIbβ3 that target site 1 (notably oral αIIbβ3 antagonists such as Lotrafiban) inadvertently activate the integrin and induce thrombosis [55]. It is possible that antagonists bind to site 2 and allosterically activate the integrin since ligand specificity to site 1 and site 2. This possibility has not been tested. Also, it would be imperative to design small molecular antagonists specific to site 2. Such antagonists will not accidentally activate this integrin.

## Figures and Tables

**Table 1:** Amino acid residues of FGF2 involved in binding to site 2 of αvβ3 (1JV2.pdb) predicted by docking simulation.

FGF2 (1BFG)	αv (1JV2)	β3
Asp-19, **Arg33**, His35, Pro36, Asp37, Gly38, **Arg39**, Val43, Glu45, **Lys46**, Ser47, Asp48, Pro49, His50, Gln56, **Lys66**, Val68, Ser69, Ala70, Asn71, **Arg72**, Tyr73, **Lys77**, **Arg81**, Leu83, Ala84, Ser85, **Lys86**, Ser87, Val88, Thr89, Asp89, Asp90, Phe93, **Lys110**	Glu15, Asn44, Gly49, Ile50, Val51, Glu52, Asn77, Asp83, Phe88, Ser90, His91, Arg122	Pro160, Val161, Ser162, Met165, Ile167, Ser168, Glu171, Glu174, Asn175, Pro186, Met187, Lys235, Val275, Gly276, Ser277, Asp278, His280, Tyr281, Ser282, Ala283, Thr285, Thr286

Amino acid residues within 0.6 nm between FGF2 and αvβ3 were selected using PDB viewer (version 4.1) (Swiss Institute of Bioinformatics, Basel, Swiss). Amino acid residues in β3 that are in the cyclic site 2 peptide are underlined.

**Table 2:** Amino acid residues of FGF1 involved in binding to site 2 of αvβ3 (1JV2.pdb) predicted by docking simulation.

FGF1 (1AXM)	αv (1JV2)	β3
Asn18, Gly19, Gly20, His21, Asp28, Gly29, Thr30, Val31, Asp32, Gly33, Arg35, Asp68, Thr69, Asp70, Leu72, Leu73, Glu81, Glu82, Lys101, Lys105, Trp107, Leu111, Lys112, Lys113, Asn114, Gly115, Ser116, Cys117, Lys118, Arg119, Pro121, Arg122, Thr123, His124, Tyr125, Gly126, Gln127, Lys128	Asn44, Gly49, Ile50, Val51, Glu52, Asp79, Ala81, Lys82, Asp83, Asp84, Pro85, Phe88, Ser90, His91, His113, Gln120, Arg122	Pro160, Val161, Ser162, Met165, Ile167, Ser168, Pro169, Pro170, Glu171, Ala172, Glu174, Asn175, Pro186, Met187, Phe188, Gly276, Ser277, Asp278, His280, Tyr281, Ser282, Thr285

Amino acid residues within 0.6 nm between FGF1 and αvβ3 were selected using PDB viewer (version 4.1) (Swiss Institute of Bioinformatics, Basel, Swiss). Amino acid residues in β3 that are in the cyclic site 2 peptide are underlined.

## Data Availability

All of the necessary data are presented in this paper. Additional data may be available upon request.

## Supplementary Materials

The following supporting information can be downloaded at: https://www.mdpi.com/article/10.3390/biom14080888/s1, Figure S1: Activation of integrin αIIbβ3 on the cell surface by FGF2.
