# Supplementary material for: FGF1 Suppresses Allosteric Activation of β3 Integrins by FGF2: A Potential Mechanism of Anti-Inflammatory and Anti-Thrombotic Action of FGF1
Source: Biomolecules. 2024 Jul 23;14(8):888. doi: 10.3390/biom14080888 (PMC11351609; doi:10.3390/biom14080888)
Supplement: Supplementary file 1 [file biomolecules-14-00888-s001.zip › biomolecules-3080196-supplementary.pdf]

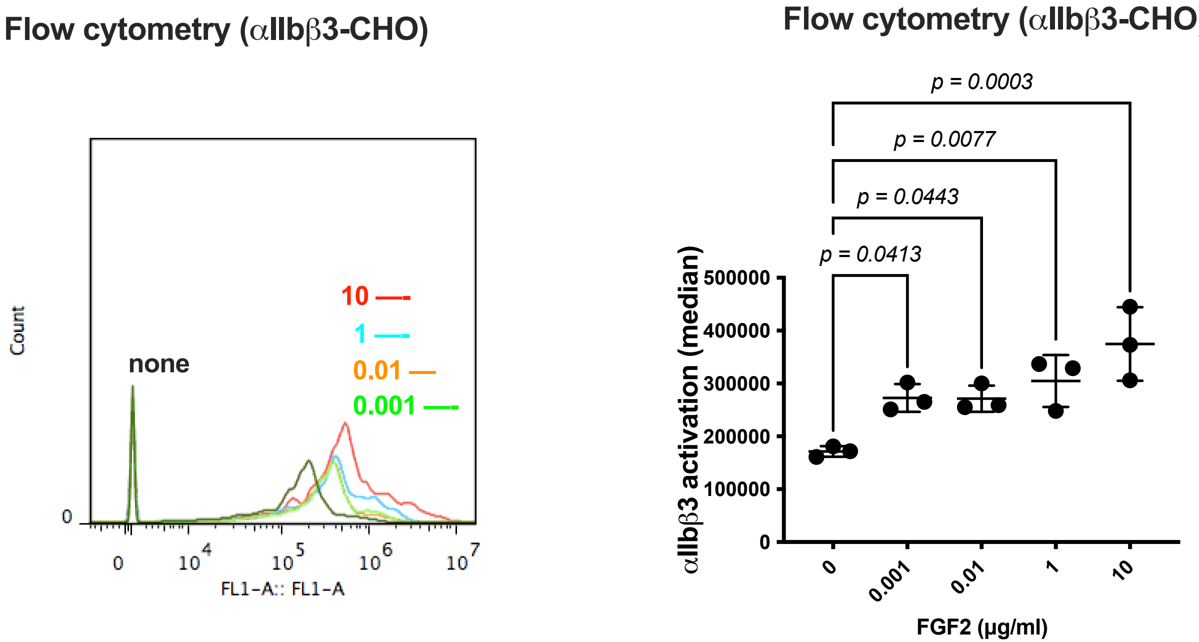

Supplemental Figure S1

Activation of integrin αIIbβ3 on the cell surface by FGF2

CHO cells that express recombinant αIIbβ3 (αIIbβ3-CHO) were cultured to nearly confluent in DMEM/10% FCS. Cells were resuspended with DMEM/0.02% BSA and incubated for 30 min at room temperature to block protein-binding sites. Cells were then incubated with WT FGF2 or mutants for 5 min at room temperature and then incubated with FITC-labeled γC390-411 for 15 min at room temperature. Cells were washed with PBS/0.02% BSA and analyzed by FACSCalibur (Becton Dickinson, Mountain View, CA). For blocking experiments, FGF2 was preincubated with Fc-β3 peptide for 30 min at room temperature.
